# 

**Published:** 1890-07-26

**Authors:** 


**A Mean and Miserable Diet** - 241

**Words of Consolation.**—Kindness Shown in Suffering - 242

**Before the Lords' Committee.** VIII.—The Nurses of the London Hospital - 243

**A New Medical Term** - 244

**The Doctor's Pulpit.**—How Suicides are Made - 245

**The History and Capabilities of Herbal Simples.**—By W. T. Fernie, M.D. XVI.—Camomile - 246

**Everybody's Page** - 247

**Annotations.**—"Ye Odde Pye"—Lepers and Penny Whistles—A Medical School for Wales - 250

**Notes and News** - 248

**The Practitioner's Mirror and Retrospect:—**

MEDICINE—Recent Views of Leprosy, 251; Gas Poisoning - 252

SURGERY—Spina Bifida - 252

NEW DRUGS, APPLIANCES, AND THINGS MEDICAL—Aërated Waters - 254

PRACTITIONERS' BOOKSHELF — London Medical Specialists 254

**Life in a London Hospital.** I. - 255

**General Charities** - 255

**Scraps and Gleanings** - 256

**Passing Topics.**—Example before Precept - 256

EXTRA SUPPLEMENT—THE NURSING MIRROR.

**En Passant.**—Letters of Thanks—Nurses Wanted—Noble Lords on Nursing Details—A Home for Epileptics—Mary Adelaide Nurses—Short Items—Trained Male Nurses - lxxi
**Sketches of Insanity.**—By Francis H. Walmsley, M.D. IV.—The Acts of the Insane - lxxii
**The Training of Nurses in France.**—Hospital Administration - lxxii
**Notes from Australia** - lxxiii
**Everybody's Opinion.**—The Nurse—A Home of Rest - lxxiii
**The Nurse** - lxxiv
**Amusements and Relaxation** - lxxiv
**Appointments** - lxxiv
**Notes and Queries** - lxxiv
**Vacancies** - lxxiv

